# 940 nm diode laser induced differentiation of human adipose derived stem cells to temporomandibular joint disc cells

**DOI:** 10.1186/s12896-022-00754-6

**Published:** 2022-08-29

**Authors:** Vesna Karic, Rahul Chandran, Heidi Abrahamse

**Affiliations:** 1 Laser Research Centre, Faculty of Health Sciences, University of Johannesburg, PO Box 17011, 2028 Doornfontein, Johannesburg, South Africa; 2 Laser Therapy in Dentistry Division, Department of Prosthodontic and Oral Rehabilitation, Health Sciences Faculty, School of Oral Health Sciences, WITS University, 7 York Street, PO Box 2010, Johannesburg, 2193 South Africa

**Keywords:** Temporomandibular disorder, Photobiomodulation, Basic fibroblast growth factor, Chondrocytes, Fibroblasts

## Abstract

**Background:**

Temporomandibular disorder (TMD) refers to a group of disorders that affect temporomandibular joint (TMJ) and its associated muscles with very limited treatment options. Stem cell research is emerging as one of the promising fields in the treatment of degenerative diseases. The ability of human adipose derived stem cells to differentiate into many cell types is driving special interest in several disease management strategies. Photobiomodulation has enhanced the role of these stem cells through their ability to promote cell proliferation and differentiation. Hence, this study examined the differentiation potential of human adipose derived stem cells (ADSCs) into fibroblasts and chondrocytes using a 940 nm diode laser for possible TMD therapy.

**Materials and methods:**

ADSCs were cultured at different seeding densities and for different time intervals. After irradiation at 24, 48, 72 h, 1, 2 and 3 weeks, ADSC viability and morphological changes were assessed in groups with and without basic fibroblast growth factor. Additionally, the level of adenosine triphosphate (ATP) in the cells was also recorded. The differentiated fibroblasts and chondrocytes were characterized with flow cytometry and immunofluorescence techniques, at 1- and 2-weeks post-irradiation.

**Results:**

Increased ATP proliferation and cell viability above 90% were observed in all post-irradiation experimental groups. Post irradiation results from flow cytometry and immunofluorescence at 1- and 2‐weeks confirmed the expression of chondrogenic and fibroblastic cell surface markers.

**Conclusion:**

This study describes stimulatory techniques utilized to differentiate ADSCs into fibroblastic and chondrogenic phenotypes using diode lasers at 940 nm. The study proposes a new treatment model for patients with degenerative disc diseases of the TMJ. The study will offer new possibilities in tissue engineering and TMJ disc management through photobiomodulation of ADSCs using a 940 nm diode laser.

## Background

The long-lasting pain condition recorded in dentistry is temporomandibular disorder (TMD). It is described as a cluster of conditions of the TMJ and its musculature. Studies have shown that approximately thirty three percent of the general population show more than one sign or symptom related to TMJ; of which only five percent of patients seek treatment [1]. In addition, the etiology of TMD is complex and has resulted in more integrative and multidisciplinary approach in diagnoses of TMD [2]. The dual axis system (Axis I is a physical assessment and diagnostic protocol and Axis II is an assessment of psychological status and pain-related disability) was established by the worldwide Research Diagnosis Criteria for Temporomandibular Disorders (RDC/TMD) with firm standards for evaluation and diagnosis of TMD globally and enhancing its importance [3, 4].

An initial approach to treat patients with TMD is occlusal splint therapy. A study has confirmed effectiveness of occlusal splint therapy in TMD patients with decline in observable pain scores [5]. Additionally, modified Jacobson’s technique as a relaxation therapy is also in practice to treat TMD [6]. Counselling and physical therapy were considered effective in the management of myofascial pain and improvement in jaw function in TMD patients [7]. However, none of these offer definitive treatment to TMD patients.

Recently, photobiomodulation has emerged as an innovative treatment for TMD patients [8]. Additionally, it has been confirmed that photobiomodulation plays multiple roles in cellular function including increased stem cell proliferation, improved immunomodulation, and tissue regeneration [9–13]. Mechanical injury to TMJ, inclusive of ischemia–reperfusion generates reactive oxygen species (ROS) in the articular TMJ tissues [14]. It has also been shown that laser light decreases this ROS generation with increase in adenosine triphosphate (ATP) in the mitochondria [15].

Over the past few decades, lasers have been in use for the treatment of musculoskeletal pain and inflammation. Studies have reported improvement in managing general TMD symptoms with 660 nm and 790 nm lasers [16, 17]. In dentistry, a study that used a 940 nm diode laser after undisplaced flap surgery delivered significantly reduced pain in patients [18]. One of the major dental root bacteria *E. faecalis* was eliminated using a 940 nm diode laser, in vitro and in vivo [19–21]. In addition, 940 nm diode laser reported decrease in post-operative trismus and swelling in patients immediately after impacted tooth extraction [22]. However, studies using this laser and stem cells in the treatment of injured TMJ disc (cells) and TMD are meagre.

Tissue engineering using stem cells is a therapeutic approach to regenerate damaged TMJ disc cells [23]. The use of biodegradable polylactide discs (PLA) with human ADSCs were found effective in the treatment of TMJ disc [24]. Only a few studies are in place defining the role of photobiomodulation promoting stem cell differentiation and therapeutic intervention [25, 26]. Also, ADSCs have shown the ability to differentiate into multiple lineages and regeneration of damaged cells [27–29]. Hence, ADSCs alone or in presence of laser could offer a promising solution in the treatment of TMJ disc degenerative changes and TMD. This therapeutic potential of ADSCs and light through differentiation to fibroblast and chondrocyte is explored in this study.

## Materials and method

### Culture of ADSCs

The Research Ethics Committee of the Faculty of Health Sciences, University of Johannesburg approved the use of immortalised ADSCs (ASC52telo, ATCC Cat # SCRC4000™ (Lot # 70003596) with a clearance number REC-241112-035. A monolayer of the ADSCs were grown in Dulbecco’s Modified Eagle Medium (DMEM, Sigma Life Science, D5796) with 10% foetal bovine serum (FBS, Gibco TM 10270 106); 1% penicillin/streptomycin (Sigma Life Science, P4333) and Amphotericin B, (Sigma Life Science, A2942). The cells were then incubated at 37 °C with 5% carbon dioxide (CO_2_) and 85% humidity.

### ADSCs differentiation into fibroblasts and chondrocytes

To induce differentiation, the cells were irradiated using 940 nm diode laser, EPIC X (SciVision Medical, BIOLASE, USA). The ADSCs cultured in a 175 cm^2^ flask were detached with TrypLE ™ Select (Gibco® 12553-029) and enumerated using an Invitrogen Countess™ II FL automated cell counter. The viable cell count was used to optimise seeding densities for all experimental groups. Cell growth, viability and morphology was observed under inverted light microscope (Wirsam, Olympus CKX41) at 24, 48, 72 h; and 1, 2 and 3-weeks. Finally, the standardized seeding densities for 24 h post-irradiation group was 5 × 10^5^, 48 and 72 h post-irradiation group—3 × 10^5^_;_ 1-week post-irradiation group 1 × 10^4^ and 2 and 3-weeks post-irradiation group—5 × 10^3^ ADSCs/3.4 cm diameter plate.

The experimental groups include control (C) with ADSCs alone (0 J/cm^2^ and no bFGF (Sigma Aldrich, Merck Group, South Africa, GF003)), LB with bFGF added (10 ng/ml) prior to the irradiation with a 940 nm diode laser, B with (10 ng/ml) bFGF only and L with laser irradiation alone. All experimental groups were incubated at 37 °C with 85% humidity and 5% CO_2_ post treatment.

### Laser setup

**Fig. 1:**
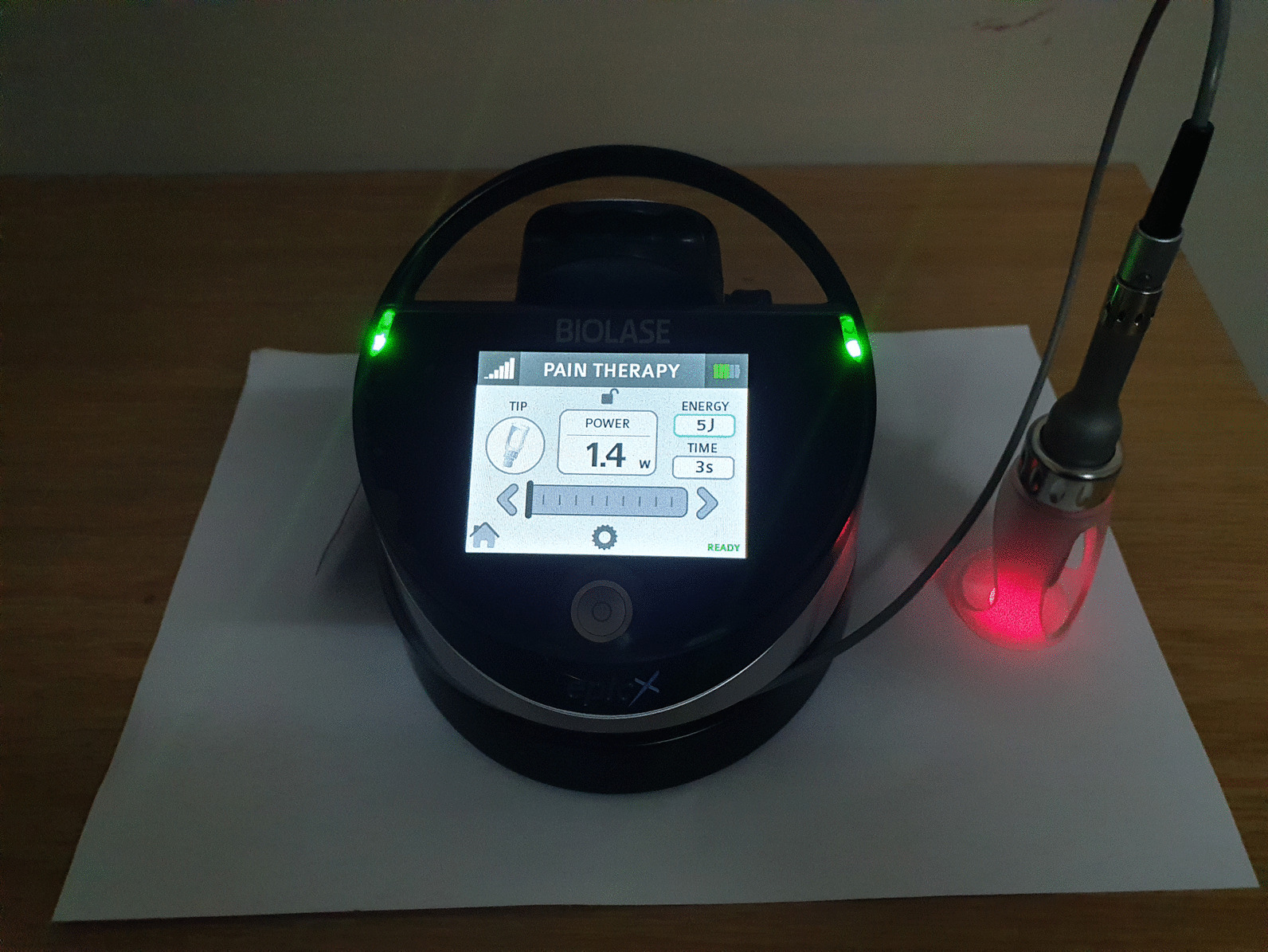
The image represents experimental settings of 940 nm diode laser, Epic X produced by SciVision Medical, Biolase, USA

A 940 nm, indium gallium arsenide phosphide (InGaAsP Semi-conductor) diode laser (Biolase, Science vision, USA), class IV; with guided red light at 1mW was used for irradiation. The ADSCs were irradiated at an energy of 5 J via TMJ handpiece in dark, adjusting the diameter of TMJ handpiece to 3.4 cm diameter plate. The power output was set at 1.4 W for all experiments with an exposure time of 3.57 s (shown as 3 s in the figure); using pain therapy settings. The irradiation parameters are shown in Table 1 and Fig. 1. The cells were then placed in an incubator with conditions mentioned above.

**Table 1:** The 940 nm diode laser parameters used for irradiation of ADSCs

Laser type	InGaAsP semi-conductor
Wavelength	940 nm
Wave emission	Continuous
Spot size	9.1 cm^2^
Output power	24, 48 and 72 h; and 1-week and 2, 3 weeks = 1.4 W
Energy	5 J
Irradiation times	24, 48 and 72 h; and 1 week and 2, 3 weeks = 3.57 s

### Assessment of cell morphology and viability

The cellular morphology of treatment groups was evaluated using an inverted light microscope (Wirsam, Olympus CKX41). Trypan blue are negatively charged, reacting only to impaired membranes of dead cells, giving visible results of the colourless viable cells [30]. Percentages of viability were recorded using trypan blue dye (Sigma‐Aldrich, Johannesburg, South Africa, T8154) mixed with equal volume of cell suspension using an automated cell counter (Countess® II FL; Invitrogen, LTC Tech South Africa Pty LTD, Fairland, Johannesburg, South Africa).

### ATP proliferation

The quantitative analyses of intracellular ATP and the mitochondrial activity indicates the incidence of energy-storing active cells as a direct indicator of cell proliferation [31, 32]. The CellTiter-Glo® 3D (Promega, Anatech Instruments, Johannesburg, South Africa) assay measures the conversion of ATP to adenosine monophosphate (AMP) by the enzyme luciferase producing luminescence. To record luminescence, equivalent volumes of reagent and ADSCs suspension (50 μl) were added to a 96 well plate (white-walled) (BD Biosciences, 353, 296). Cell lysis was initiated by placing the reaction mixture on a shaker for a duration of 5 min. After incubation in dark for 25 min, luminescence was measured in relative light units (RLU) using Victor 3 multiplate reader (Perkin-Elmer, Johannesburg, South Africa). The experiments (C, LB, B and L) were repeated three times.

### Flow cytometry analysis

Based on the preliminary experimental results from above, the percentage differentiation of ADSCs into fibroblasts and chondrocytes at 1- and 2-weeks was observed post- irradiation with 940 nm diode laser at 5 J. Fibroblast marker CD26 (clone M-A261, mouse anti human, Bio Rad Laboratories (Pty) Ltd, South Africa) and chondrocyte marker CD49C (clone P1B5, mouse anti human, Bio Rad Laboratories (Pty) Ltd, South Africa) were used to confirm differentiation in all the experimental groups.

The cells detached from culture flasks were centrifuged at 400 g for 5 min and re-suspended in 1 ml of phosphate buffered saline (PBS) (A2153 and S8032; Sigma, Johannesburg, South Africa) at ambient temperature and an automated cell counter was used to establish cell count and viability. Thereafter, 1 × 10^6^ cells were added into a focus tube in 100 μl PBS at 4 °C followed by10μl of primary antibody and vortexed.

After incubation in dark for 30 min, the cells were rinsed three times using PBS and centrifuged at 400 g for 5 min at ambient temperature. The cells were then labelled with 10 μl of the secondary antibody (FITC Goat anti‐Mouse; Santa Cruz Biotechnology, Anatech Instruments, Johannesburg, South Africa) and incubated in dark for 30 min. After rinsing with PBS, the antibody labelled cells were centrifuged at 400 g for 5 min at ambient temperature. Finally, cells were re-suspended in 300 μl of PBS for the instant flow cytometry reading. The Accuri C6 flow cytometer (BD Biosciences, Ascendis Medical, Johannesburg) was used to establish the presence of differentiated cells with fluorescence (dye-stained cells). The FL-1 filter at 533/30 and 488 nm laser was used for the analysis.

### Immunofluorescence

The best recognised among the methods of immunofluorescence are indirect and complement binding [33, 34]. The differentiated ADSCs to fibroblasts in L, LB and B groups at 1- and 2-weeks post-irradiation were confirmed through indirect immunofluorescence protocol in the current study. The differentiated chondrocytes in L group were observed at the same time interval using the same protocol.

The cells were cultured on heat sterilized coverslips in 3.4 cm diameter culture dish with 2 ml of complete media at a concentration of 1 × 10^4^ for 1-week and at 5 × 10^3^ of cells/plate for 2-weeks after irradiation. The ADSCs in experimental groups were rinsed with ice cold PBS (Sigma, A2153), and fixed in 4% paraformaldehyde (Sigma, P6148), incubated in dark for 15 min. Subsequently, the blocking solution (10% (w/v) BSA (bovine serum albumin) was added to the cells and incubated for another 30 min at room temperature. After washing three times with ice cold PBS, 100 μl primary antibody CD26 (mouse anti human; 1:100 μl of PBS dilution) was added to the cells and incubated for 1 h. Later, washing the cells with PBS was repeated. Thereafter, the cells were labelled with 100 μl of the secondary fluorescent FITC Goat anti-mouse antibody (1:100 μl of PBS dilution) and incubated for another 1 h in dark.

The labelling of CD49C marker (1: 200 μl of PBS dilution) was performed as done for CD26. Finally, ADSCs nucleus were counterstained with 4′-6-diamidino-2-phenylindole (DAPI) ((Invitrogen™, D1306) 358Ex/461Em). After 10 min incubation, coverslips were placed on glass slides and mounted using Fluoromount™ Aqueous Mounting Medium in dark (Sigma, F4680). A fluorescent microscope live cell station from Carl Zeiss Axio Z1 Observer using AxioVision imaging software (Carl Zeiss, Randburg, Johannesburg, South Africa) was used to record images.

### Statistical analyses

All the results were expressed as mean ± SEM (n = 6). ANOVA statistical analyses was performed using Sigma Plot version 13.0. *p* < 0.05 was considered statistically significant. The statistical significance results were plotted on the graph with *p < 0.05, **p < 0.01, ***p < 0.001.

## Results

With the series of experiments performed above in the present study interesting results were obtained. All the protocols were for differentiation proceeded with the inference obtained from the morphological and biochemical assays beyond 72 h.

### Cell morphology and viability

**Fig. 2:**
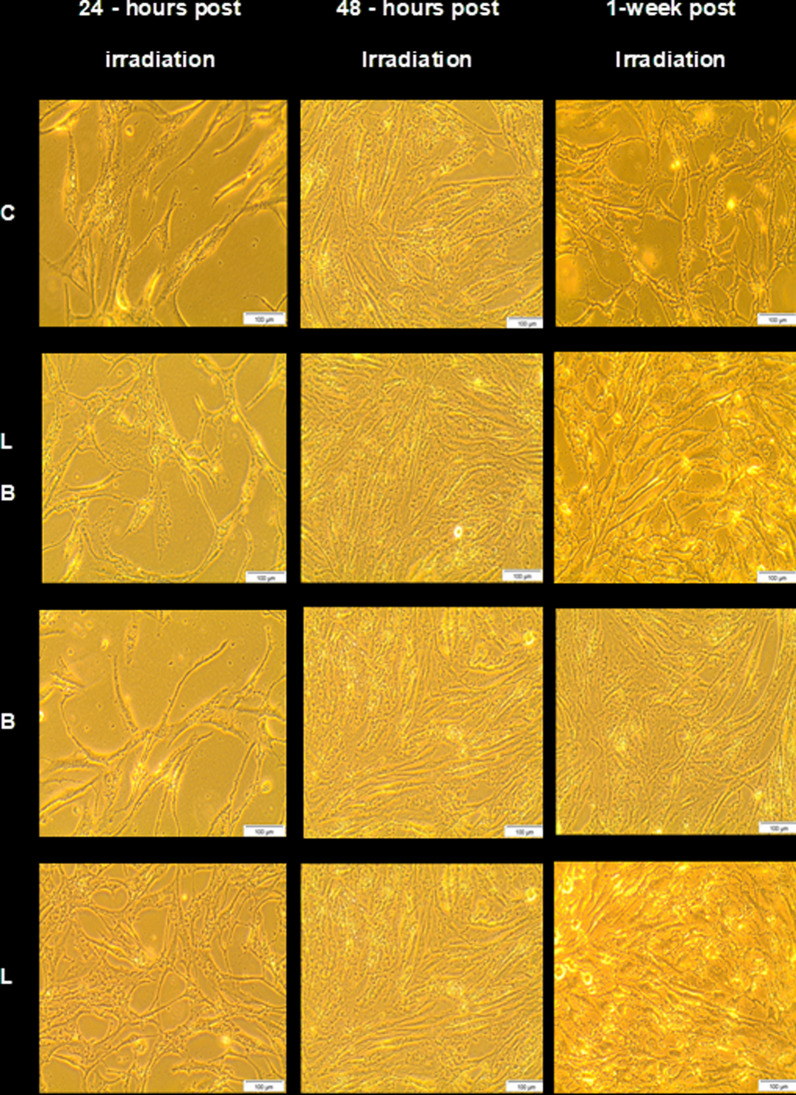
Cellular morphology of ADSCs assessed at 24, 48 h and 1-week post-irradiation with 940 nm diode laser. The cells appear viable with no signs of cell death and have clearly proliferated beyond 72 h in laser and bFGF groups

The images captured in the study after treatment were observed for evident changes in cell morphology, proliferation or death. Post-irradiation morphological images confirmed proliferation at 24, 48 and 72 h; and 1-, 2- and 3-weeks. The confluence and morphology depicted healthy cells in all experimental groups. Beyond 72 h, all experimental groups with/without laser irradiation and bFGF did not show much change in cell proliferation. Hence, images showing significant proliferation changes at different time intervals are only shown in Fig. 2.

**Fig. 3:**
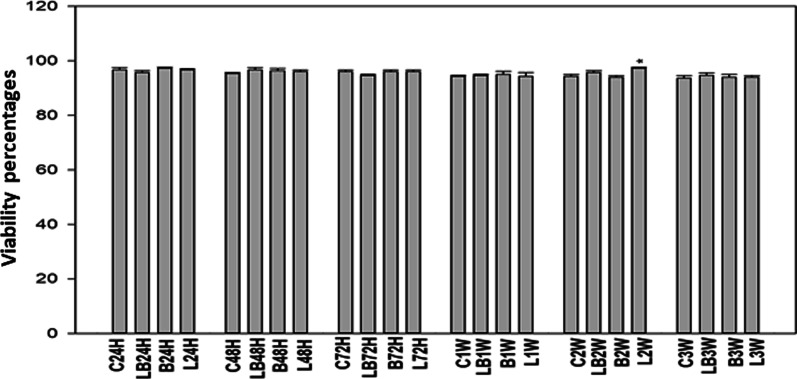
Post irradiation viability assessment of ADSCs using trypan blue at different time-intervals with 940 nm diode laser. The groups show similar to no difference in in groups with above 90% viable cells. The only statistical significance (*p* < 0.05) recorded with student’s one tail t-test can be seen at 2-weeks, in laser treated group. The statistical significance is presented as *p < 0.05

All post-irradiation experimental groups with/without bFGF showed high viability. The highest percentage of viable cells are presented in group L at 2-weeks with a statistical significance of *p* < 0.05 (Fig. 3).

### ATP proliferation

**Fig. 4:**
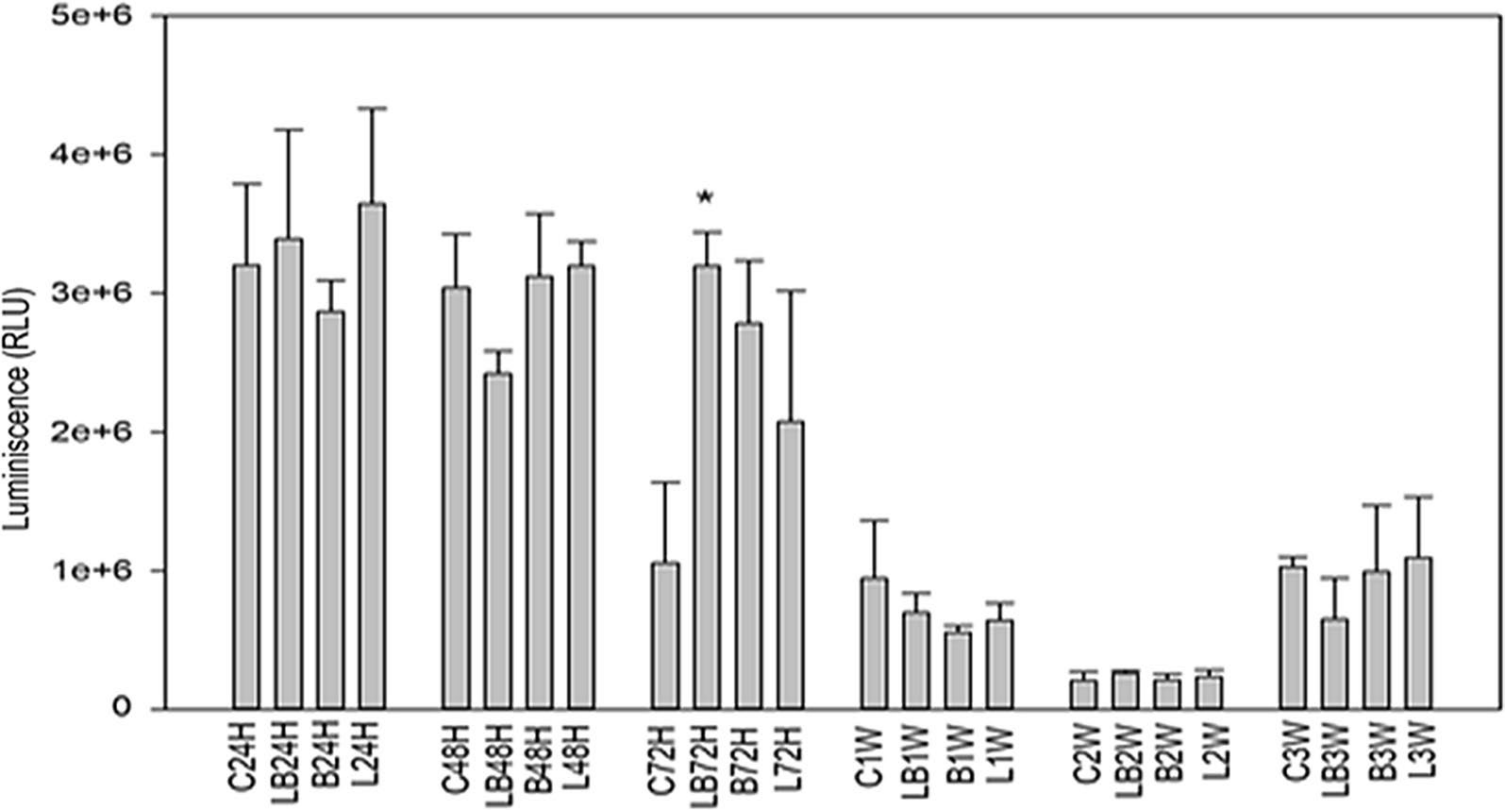
Cellular ATP proliferation assessed at 24, 48 and 72 h: and 1-week and 2,3 weeks post-irradiation with 940 nm diode laser. The ATP proliferation was evident in all experimental groups. The importance of bFGF and laser in cell proliferation and an increase in ATP is clearly reflected. A significant increase in ATP compared to cells in other groups is evident at LB72 h. The statistical significance is presented as *p < 0.05

The results of ATP proliferation was important to support the proliferation and cellular viability. The experimental groups for ATP proliferation were compared within the groups, to their respective controls due to different seeding densities. Although the viability results supported ATP proliferation, the only statistical significance was verified in the LB group at 72 h post-irradiation (Fig. 4).

### Flow cytometry analyses

**Fig. 5:**
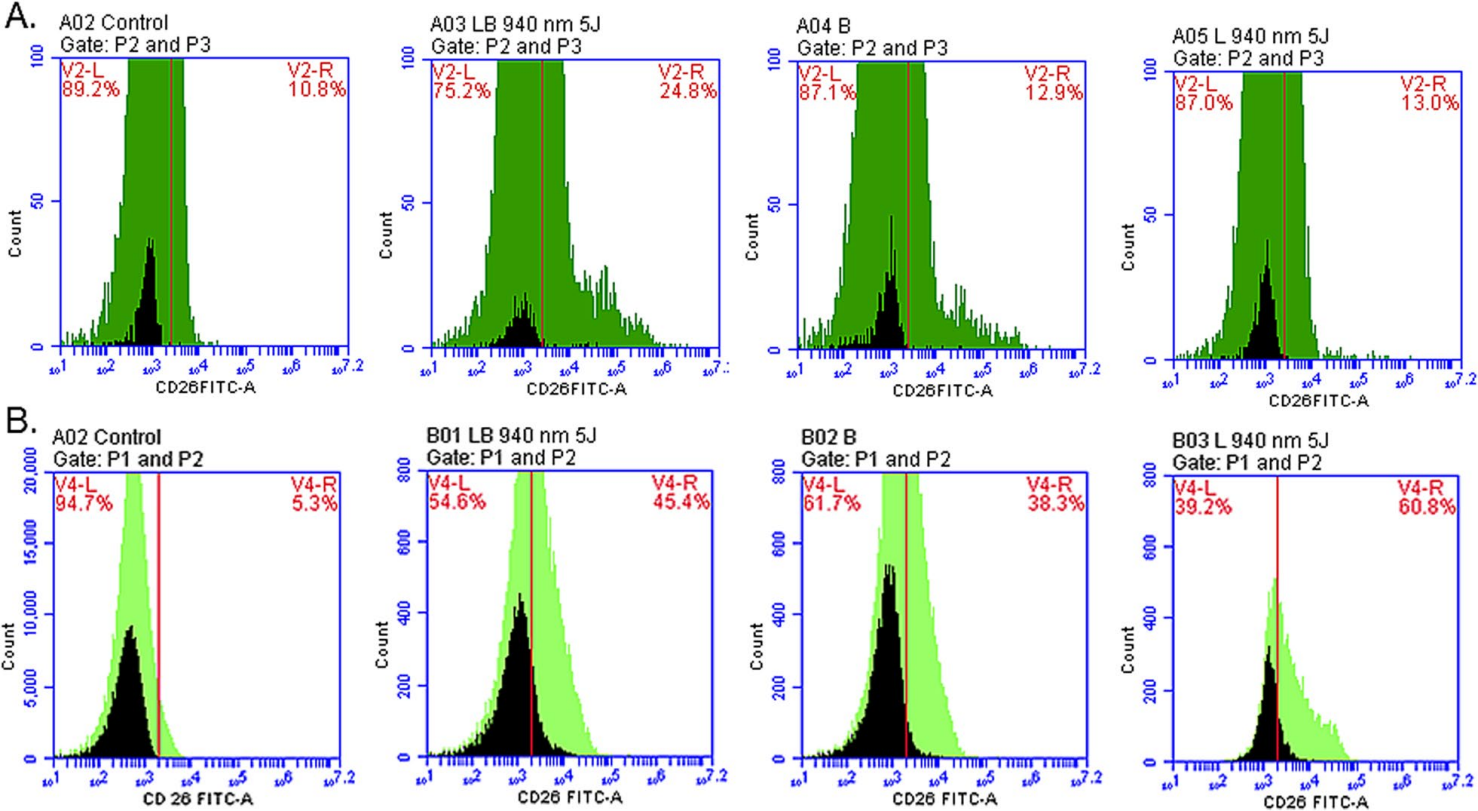
Positive expression of CD26 presented by flow cytometry with 940 nm diode laser. **A** Expression of CD26 at 1-week post-irradiation presenting percentages of differentiation in all experimental groups. The percentage of expression was higher in LB group at 1-week and **B** at 2-weeks post-irradiation and group L had the highest expression of CD26 marker compared to control group with no markers

**Fig. 6:**
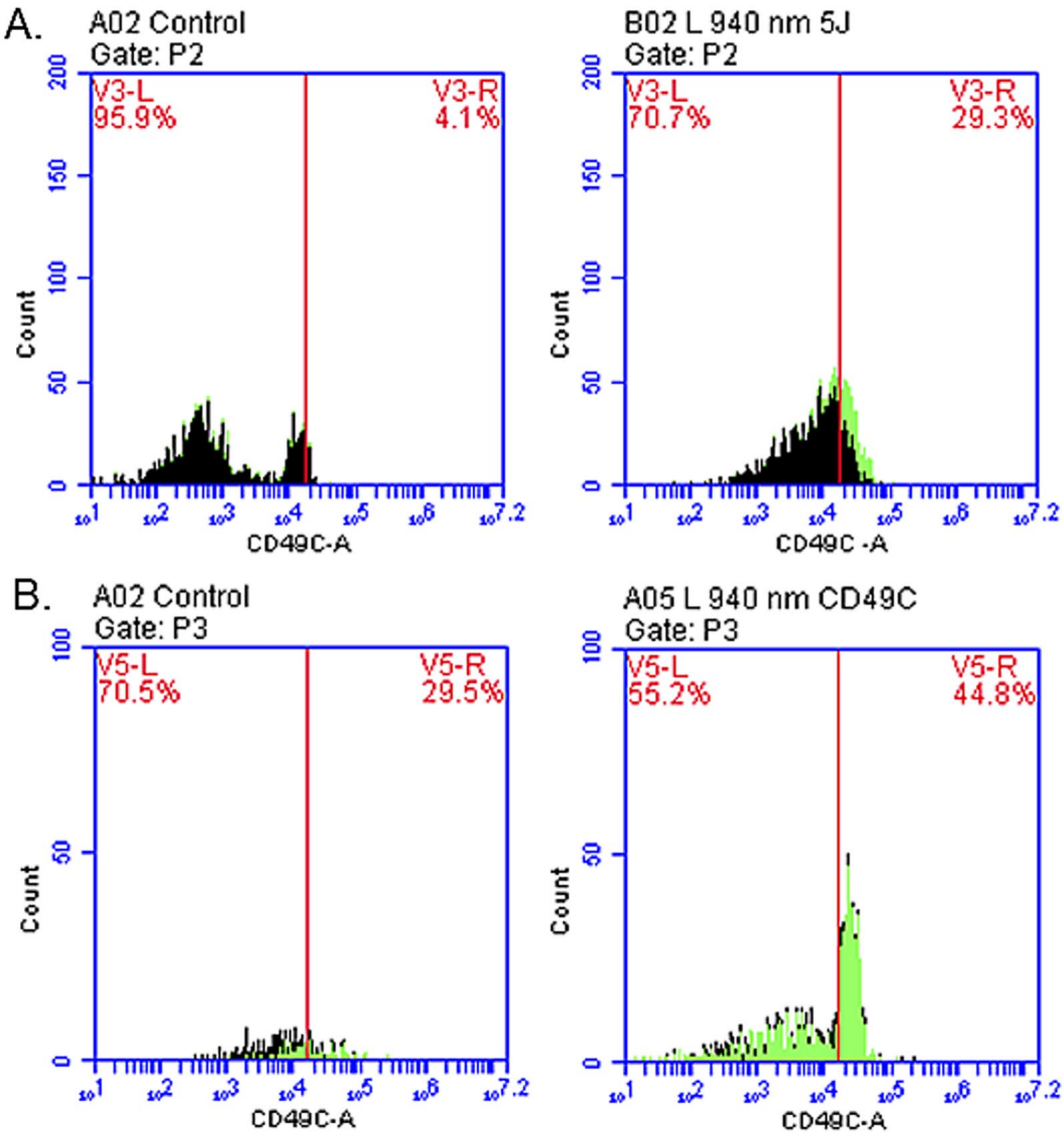
Positive expression of CD49C marker recorded with flow cytometry with 940 nm diode laser. The expression of CD49C marker was evident at 2-weeks’ post-irradiation, **B** than at 1-week **A** when compared to control groups (no irradiation and no markers added)

The surface marker CD26 for fibroblasts expression was observed in LB, B, and L groups at 1-week and 2-weeks after irradiation. In other groups no to minimal signs of differentiation was observed. The highest percentage (60.80%) of expression was recorded in L group, at 2-weeks after irradiation. The groups with percentages of differentiation are presented in Fig. 5A, B, Table 2. Furthermore, compared to control, 29.30% chondrocytes at 1-week and 44.8% at 2-weeks post-irradiation were detected in the laser only group (Table 2) (Fig. 6A, B).

**Table 2:** Flow cytometry results of experimental groups

Flow cytometry experimental groups			
	B	LB	L
CD26 at 1-week post-irradiation	8.97% ± 2.08**	16.57% ± 2.8**	8.67% ± 2.2*
CD26 at 2-weeks post-irradiation	19.20% ± 9.58	20.83% ± 12.3	45.00% ± 8.3*
CD49C at 1-week post-irradiation			13.20% ± 8.1
CD49C at 2-weeks post-irradiation			38.03% ± 0.0

The experimental groups of cells are labelled as follows: LB—laser irradiation and bFGF, B—bFGF only and L—laser irradiation only. The statistical significance is presented as *p < 0.05, **p < 0.01

### Immunofluorescence

**Fig. 7:**
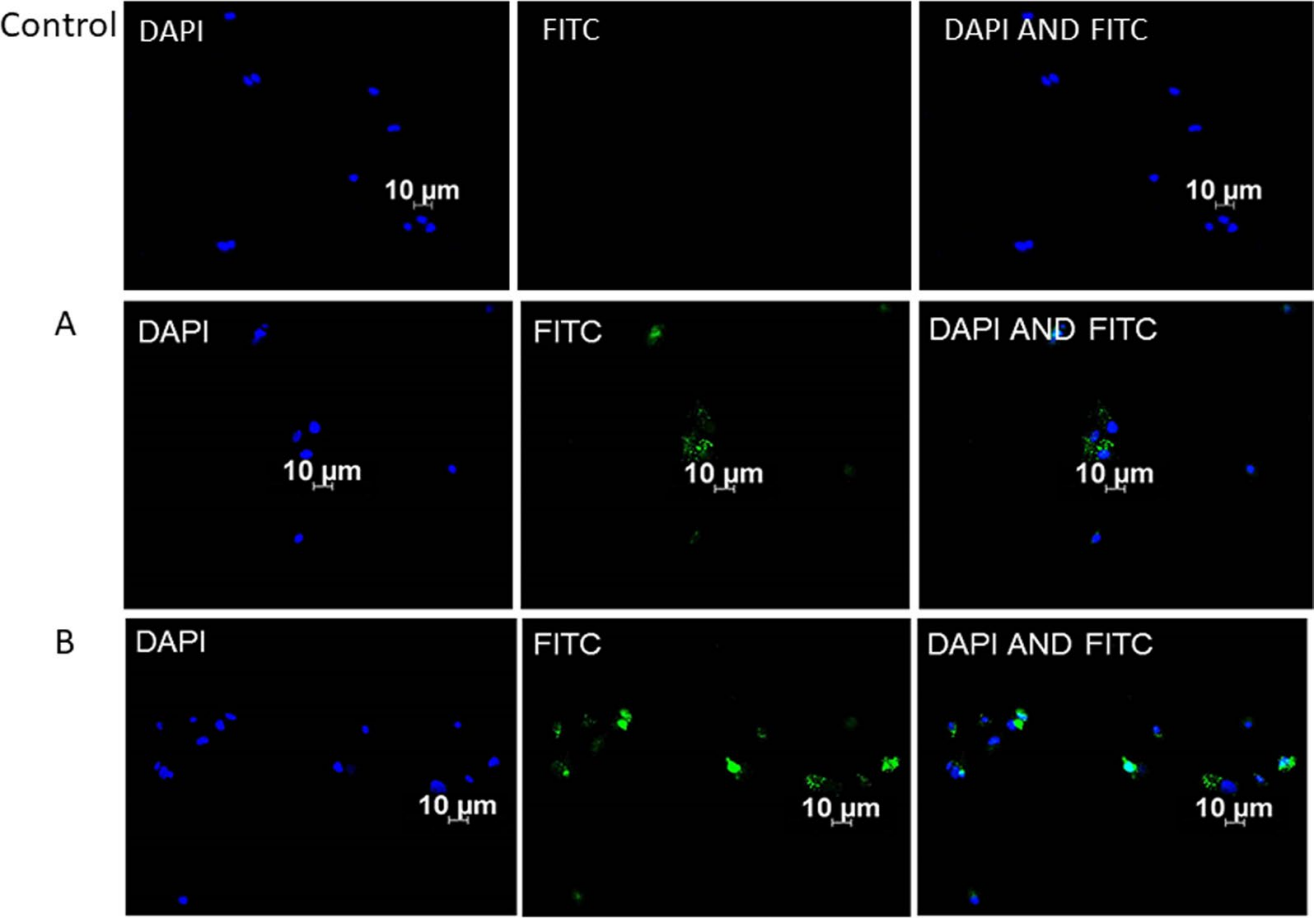
Differentiation of ADSCs to fibroblasts with 940 nm diode laser confirmed through immunofluorescent microscopic images. The differentiation of ADSCs to fibroblasts in group L at 1-week (**A**) and 2 weeks (**B**) post-irradiation presented as a green fluorescence (FITC) represents the expression of CD26. The nuclear counterstaining DAPI is represented in blue colour. The fluorescence and expression of marker on treated (**A** and **B**) cells are compared to untreated control ADSCs with no signs of differentiation

**Fig. 8:**
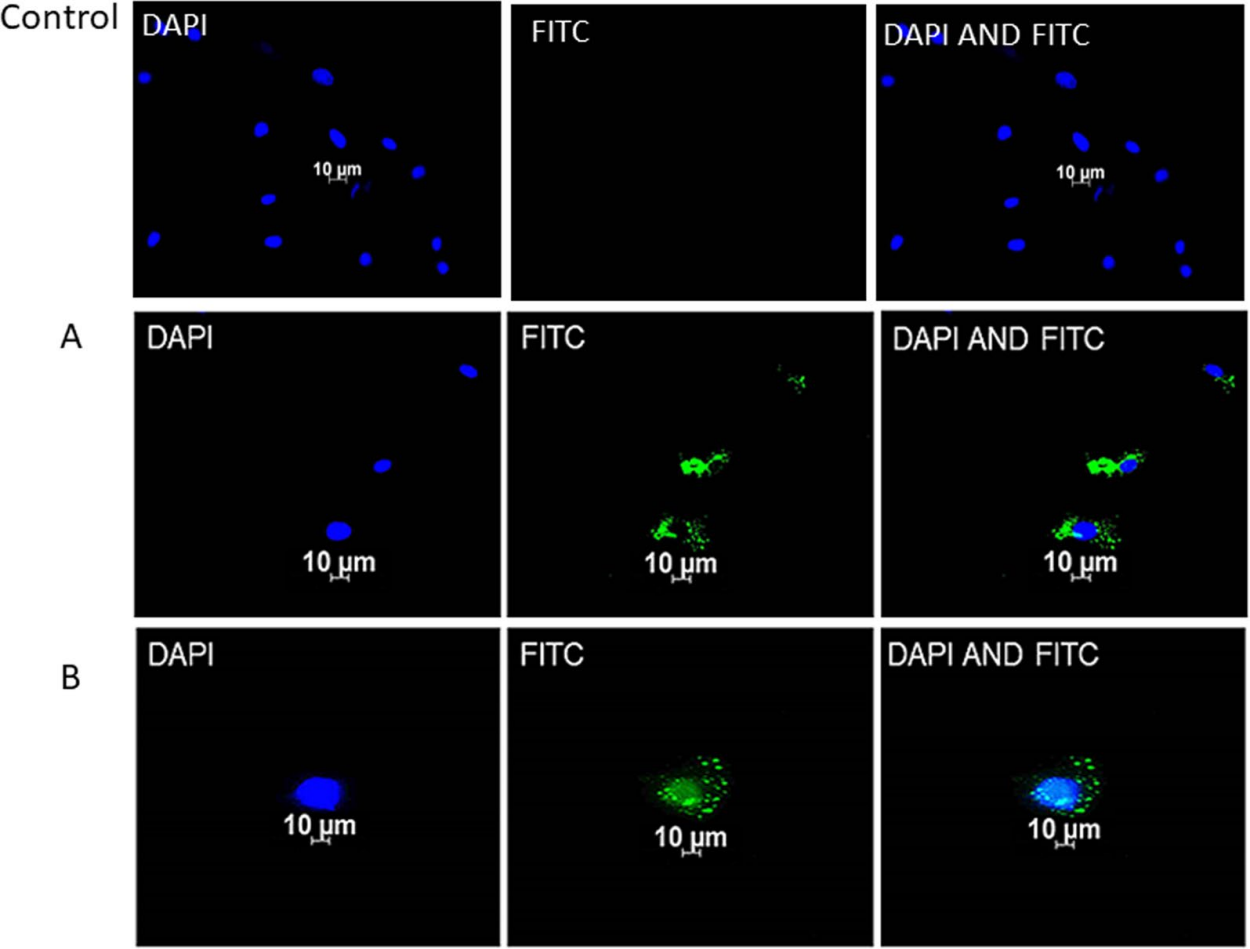
Immunofluorescent microscopic images confirming the differentiation of ADSCs to chondrocytes post-irradiation with 940 nm diode laser. The green, fluorescent stain (FITC) signifies expression of CD49C in images, endorsing the differentiation of ADSCs to chondrocytes in group L at 1-week (**A**) and at 2-weeks (**B**) post-irradiation. With no FITC fluorescence in control groups, the chondrocyte differentiation induced by laser is clear in (**A** and **B**)

As observed in flow cytometry, differentiation of ADSCs into fibroblasts was analysed at 1-week and 2-weeks in LB, B, and L experimental groups. Figure 7A, B shows images of surface marker (CD26) expression and differentiation with FITC green fluorescence in above mentioned experimental groups. Also, the differentiation of ADSCs into chondrocyte was observed through fluorescent expression of CD49C marker in group L at 1-week and 2-weeks (Fig. 8A, B).

## Discussion

This study reports application of 940 nm diode laser at 5 J in differentiating ADSCs into functional fibroblasts and chondrocytes beyond 72 h for the first time.

The clinical application of a 940 nm diode laser and other lasers in dentistry has been on increase. A study that used a 940 nm diode laser has reported significant reduction of pain in TMD patients [35]. Another study reported an ease in jaw movement after application of 830 nm laser in TMD patients [36]. Further evidence from a study that used a 808 nm laser has shown reduced physical symptoms in TMD patients with/without pain [37]. Nevertheless, only few studies have explored the effect of 940 nm diode laser on cellular level.

The current study has established the proliferative effect of 940 nm diode laser at 5 J on ADSCs beyond 72 h. A similar seeding density for different groups would result in multi-layered overgrowth and cell death. Hence, different seeding densities were used throughout the study. An increase in proliferation with a high viability was confirmed in all experimental groups. A finding that relates to current study results was reported in diabetic wounded fibroblasts at 48 and 72 h, where cell migration, viability, proliferation, and collagen content significantly increased after irradiation with 660 nm diode laser (5 J) [38, 39]. Our results offer convincing evidence on how this laser can initiate possibility of differentiation for curative applications of 5 J against degenerative TMJ disc disorder.

Further on, we also noticed that the ATP proliferation did not show an evident increase like in other groups with laser and bFGF as observed in 1- week and 2- weeks. This could be an indication of cells entering into differentiation phase. To further investigate, flow cytometry and immunofluorescence studies were performed to detect signs of possible differentiation of ADSCs to fibroblasts and chondrocytes in the above experimental groups. As a result, the experiments confirmed differentiation of ADSCs to fibroblasts in groups treated with bFGF using CD26. A comparable result was reported by a study where a 940 nm diode laser at time intervals of 24 and 72 h had a stimulating effect on fibroblasts without shifting the cell cycle; with elevated proliferative capacity and cell differentiation [40].

Moreover, a statistical significance was noted in flow cytometry analyses for fibroblasts at 1-week post-irradiation in B (8.97%) and LB group (16.57%). Similar results were published with 660 nm diode laser with statistically significant results in same experimental groups at 2-weeks post-irradiation [41]. Hence, the current study results also indicate possible application of lasers (940 nm and 660 nm) induce differentiation of ADSCs into fibroblasts. This could be achieved with/without bFGF and at different time intervals giving promising novel option for the better management of degenerative TMJ disc.

The differentiation of ADSCs to chondrocytes was done without the growth factor in the current study, due to a previous known fact from a study that reported bFGF at 10 ng/ml concentration employs an inhibitory effect on osteoblast differentiation [42]. Hence in this study, only laser irradiation was applied without the use of bFGF. Also, a related study used lasers only to differentiate MSCs into bone or cartilage (660 nm, 485 nm, 810 nm, 532 nm) [43]. Additionally, immunofluorescent results of the current study have established the expression of CD49C marker in laser group only at 1-week and 2-weeks post-irradiation.

940 nm diode laser has also shown to stimulate human foetal osteoblast differentiation to improve bone formation [44]. Nevertheless, comparing the previously published results of 660 nm, chondrocytes differentiation was better observed at 2-weeks post-irradiation than with 940 nm diode laser. These outcomes indicate the significance of time interval of incubation and wavelength of irradiation as a crucial factor in differentiation of ADSCs to chondrocytes. Hence, the results recommend application of lasers at different wavelengths and time intervals.

Additionally, the current experimental results with 940 nm diode laser points to the fact that the lasers alone can be used in regenerative treatment of degenerative of TMJ disc. These results relate to a study that accelerates soft tissue regeneration and bone formation with 940 nm diode laser in vitro [45]. Furthermore, a combined application of 940 nm at 5 J and nanomaterials on ADSCs offers promising option for tissue regeneration in TMJ disc, allowing novel transplantation process [46–48].

Likewise, laser irradiation on its own with 940 nm at 10 J/cm^2^ fluence was reported to stimulate a healing effect on palatal mucoperiosteal wounds with possible production of fibroblasts [49]. Another study reported that human periodontal ligament stem cells (hPDLSCs) seeded on calcium phosphate cement (CPC) scaffolds were able to differentiate into the osteogenic cells enhancing bone regeneration in dental, craniofacial, and orthopaedic applications [50]. In a different study the hPDLSCs were differentiated into osteoblasts, fibroblasts and cementoblasts, for better regeneration of periodontium [51]. From these reports and the results from the current study, application of 940 nm diode laser on ADSCs could improve prospects of tissue engineering for degenerative TMJ disc provided that the constitution and function; and inflammatory mediators are researched intensely [52–56]. In addition, future studies with the ADSCs and nanolaser as part of nanorobotics application in dentistry could enhance the therapeutic role in the clinical management of degenerative changes of the TMJ disc cells [57]. Nevertheless, the novel findings of the current study will promote future research in this field.

## Conclusion

Convincingly, in the current study substantial results from ATP proliferation, flow cytometry, and immunofluorescence studies beyond 72 h post-irradiation with 940 nm diode laser have been established and the differentiation of ADSCs towards fibroblastic and chondrogenic phenotypes were confirmed. The presence and absence of bFGF and role of 940 nm laser in the differentiation is clearly stated in the study. Time interval was crucial in this study as the differentiation was observed at 1- and 2-weeks post irradiation compared to other groups. The recommendation is to explore a wider range of laser wavelengths, dosages, possibility of laser combinations, time of irradiation and gene expression studies. This observation will include a more profound understanding of biochemical processes of TMJ disc. The outcomes of the current study will direct future studies at preclinical and clinical levels in the replacement of damaged TMJ disc cells with nanolasers and offer new opportunity in improving curative management of the degenerative TMJ disc.

## Data Availability

All data generated or analysed during this study are included in this published article.

## Abbreviations

TMD: Temporomandibular disorder
TMJ: Temporomandibular joint
ADSCs: Adipose derived stem cells
bFGF: Basic fibroblast growth factor
ATP: Adenosine triphosphate
RDC/TMD: Research Diagnosis Criteria for Temporomandibular Disorders
PLA: Polylactide
DMEM: Dulbecco’s Modified Eagle Medium
FBS: Foetal bovine serum
CO_2_: Carbon dioxide
InGaAsP: Indium gallium arsenide phosphide
RLU: Relative light units
AMP: Adenosine monophosphate
PBS: Phosphate buffered saline
ATCC: The American Type Culture Collection
